# Prevalence and perceived barriers to physical activity among healthcare students: a cross-sectional study in the West Bank

**DOI:** 10.3389/fpubh.2026.1937884

**Published:** 2026-10-07

**Authors:** Maaweya Jabareen, Wasef Alhroub, Ahmad Fasfoos, Fatima Hamad Thabata, Isra Hamoudah, Hala Hasasneh, Malak Al-Ayaseh, Ayham R. A. Zidat, Sama R. Misk, Esraa Jaber, Abdallah Alwawi

**Affiliations:** 1 Faculty of Medicine, Hebron University, Hebron, Palestine; 2 Faculty of Medicine, Al-Quds University, Jerusalem, Palestine; 3 Faculty of Medicine, Palestine Polytechnic University, Hebron, Palestine; 4 Department of Nursing, Faculty of Health Professions, Al-Quds University, Jerusalem, Palestine; 5 INTI International University, Nilai, Negeri Sembilan, Malaysia

**Keywords:** barriers to physical activity, exercise patterns, healthcare students, physical activity, sedentary behavior

## Abstract

**Background:**

Physical activity (PA) contributes to disease prevention, mental wellbeing, and overall quality of life. Despite these benefits, students in demanding academic tracks, such as medicine and allied health, often struggle to remain active. Evidence on PA patterns among healthcare students in Palestine is limited, although this group is expected to model healthy behaviors in their future practice. This study set out to examine how frequently students engage in PA, what forms it takes, and the barriers they encounter.

**Methods:**

An analytical cross-sectional study was conducted between February and July 2025 among 408 undergraduate healthcare students from four universities in the West Bank. Data were collected using an Arabic questionnaire adapted from the WHO Global Physical Activity Questionnaire (GPAQ). Physical activity was classified as low, moderate, or high according to total MET-minutes/week. Associations with sex, academic year, and healthcare specialty were assessed using appropriate statistical tests. Multivariable multinomial logistic regression was performed with high physical activity as the reference category.

**Results:**

The primary finding was that 68.1% of healthcare students were classified as having high physical activity, while 22.5% had moderate and 9.3% had low physical activity according to WHO GPAQ criteria. Overall, 90.7% had moderate or high physical activity. Physical activity level differed significantly across healthcare specialties (*p* < 0.001), with medical students having lower total physical activity than nursing and physical therapy students in post-hoc comparisons. Healthcare specialty remained independently associated with physical activity level in multivariable analysis (*p* < 0.001), whereas sex and academic year were not. Although 76.5% reported engaging in at least one type of physical activity during a typical week, this measure differed from GPAQ-based classification. Lack of time was the most commonly reported challenge (65.4%). Males had higher total MET-minutes/week than females (*p* = 0.033), although sex was not associated with physical activity category.

**Conclusion:**

Most healthcare students had moderate or high PA levels, although substantial variation was observed across healthcare specialties. Medical students showed comparatively lower PA than several other specialty groups, while lack of time remained the most frequently reported challenge. These findings support specialty-sensitive strategies that facilitate physical activity within demanding academic schedules.

## Background

Physical activity (PA), defined by the World Health Organization (WHO) as any skeletal muscle movement that expends energy, is a critical determinant of physical and mental health (1). Beyond total activity volume, increasing attention has been given to physical activity patterns, which describe the temporal structure and distribution of physical activity and sedentary behavior accumulated over a specified period of time (2). A recent review confirmed that high levels of occupational PA are associated with a reduced risk of various cancers, coronary heart disease, and type-2 diabetes, while also improving mental health and enhancing overall well-being and quality of life (3, 4).

Evidence indicates that PA significantly affects the health of both adults and children (5), yet one in five adults globally remains physically inactive, with higher prevalence in wealthier, urban populations, as well as among women and older adults (6). Physical inactivity has been identified by the WHO as a major modifiable risk factor for morbidity and mortality, alongside smoking, obesity, and hypertension (7).

University students are a population of particular concern, as physical activity has been associated with improved academic performance and cognitive function (8, 9), Nevertheless, many students fail to meet recommended activity levels due to academic workload, part-time jobs, and social commitments (9, 10).

PA is typically classified into three main intensities. Vigorous-intensity activities are those that require substantial physical effort and result in a significant increase in heart rate, reaching approximately 70 to 85% of the maximum heart rate (11). Moderate-intensity activities require a moderate amount of physical effort and cause a noticeable but smaller increase in breathing or heart rate, typically reaching about 50 to 70% of the maximum heart rate (11). Sedentary behavior is defined as any waking behavior characterized by an energy expenditure of ≤1.5 metabolic equivalents (METs) while in a sitting, reclining, or lying posture (12).

The WHO recommends that children and youth (15–17 years) engage in at least 60 min of mostly aerobic activity daily, including muscle- and bone-strengthening exercises three times a week. Adults (18–64 years) should engage in at least 150–300 min of moderate or 75–150 min of vigorous aerobic activity weekly, plus muscle-strengthening exercises ≥2 days/week (13). The WHO Global Physical Activity Questionnaire (GPAQ) provides standardized criteria to classify individuals as active or inactive, with inactivity defined as <600 MET minutes/week of moderate-to-vigorous activity or equivalent patterns (14).

Healthcare students, particularly those in medicine and nursing, may face challenges in maintaining adequate levels of PA (15, 16). Healthcare students also face additional challenges, including intense academic demands, study-related activities, and extended shift work, with lack of time frequently reported as a barrier (17, 18).

Other factors contributing to physical inactivity among university students in general include mental stress, irregular sleep, limited access to exercise facilities, financial constraints, socio-cultural factors (especially affecting females), environmental safety concerns, and low motivation or awareness (19).

The majority of PA studies have been carried out in developed countries, resulting in a notable lack of research in low- and middle-income nations (20). A 2012 study in Palestine reported that men were more physically active than women, primarily through work and domestic activities, while women engaged less in leisure-time activity and walking. Overall, 26% of women were classified as insufficiently active compared to 13% of men, with inactivity particularly high among older, less-educated, and unemployed or retired individuals (21). Despite these findings, no studies have examined the prevalence and barriers to PA among university students, particularly healthcare students, who face unique academic, clinical, and sociocultural challenges. These factors may influence both awareness of recommended PA guidelines and actual engagement in PA. To address this gap, the present study aims to investigate the prevalence, patterns, and determinants of PA among healthcare students in Palestine.

## Methodology

This cross-sectional analytical study was conducted between February and July 2025 among undergraduate healthcare students from four major universities in the West Bank, Palestine: Hebron University, An-Najah National University, Palestine Polytechnic University, and Al-Quds University.

The required sample size was calculated using the standard formula for cross-sectional studies, assuming an expected prevalence of 50%, a 95% confidence level, and a 5% margin of error, yielding a minimum required sample size of 384 participants. A total of 408 students aged 18 years or older—the typical minimum age for university admission—were recruited through convenience sampling using institutional emails and social media platforms. Because the questionnaire was distributed through multiple electronic channels and the number of students who received the invitation could not be reliably determined, a response rate could not be calculated. Eligibility required active enrollment in a healthcare program at these universities, while exclusion criteria included incomplete surveys, pregnancy, and enrollment in programs outside the healthcare field.

Complete case analysis was applied to the dataset. Of the 424 questionnaires collected, 16 (3.8%) were excluded due to incomplete PA data. We prioritized complete GPAQ responses to ensure accurate classification of PA levels, which was the main focus of the study. Minor missing responses in demographic variables were retained when not required for a specific analysis, as they did not affect the assessment of the primary outcome, and the low proportion of missing responses supports the appropriateness of this approach while maintaining the validity of the study results.

Data were collected using a validated, self-administered Arabic questionnaire adapted from the World Health Organization’s GPAQ, with additional sections added based on an extensive review of similar published studies (22, 23).

The GPAQ allows standardized assessment of PA and compliance with the WHO PA Guidelines and is implemented in more than 100 countries as part of the WHO Stepwise approach. The instrument contains 16 questions that capture duration, intensity, and frequency of activity across three domains: occupational, transport-related, and leisure-time PA. The final translated Arabic version of the questioner was reviewed by a panel of three experts with backgrounds in public health, family medicine, and biostatistics, all of whom had prior experience in research methodology. Based on their feedback, minor revisions were made to improve clarity, simplify terminology, and ensure that all items were easily understood by students. The questionnaire was then pilot-tested among 30 students to assess clarity and cultural relevance. The final instrument comprised four sections: demographic characteristics, PA across (work, transport, and leisure domains), sedentary behavior, and self-reported barriers to PA.

Both the demographic section—including age, sex, academic year, field of study, place of residence, and engagement in weekly PA—and the assessment of challenges and barriers to PA were adapted from a previously published study, with minor modifications made by the expert panel, such as the addition of the place of residence item (22, 23). Potential barriers to physical activity were assessed among all participants, regardless of their current weekly physical activity status. Barrier responses were summarized for the total sample and descriptively stratified according to self-reported weekly physical activity (Yes/No).

GPAQ responses were scored using standard MET coefficients, assigning 8 METs to vigorous-intensity activity and 4 METs to moderate-intensity activity and active transport. Total MET-minutes/week was calculated as the sum of MET-minutes accumulated across all GPAQ activity domains. For the three-level PA outcome used in the analyses, participants were classified as having low (<600 MET-min/week), moderate (600–3,000 MET-min/week), or high (>3,000 MET-min/week) physical activity.

Data were analyzed using IBM SPSS Statistics version 27. Categorical variables were summarized as frequencies and percentages, while non-normally distributed continuous variables were presented as medians and interquartile ranges (IQRs). Associations between categorical variables were assessed using Pearson’s chi-square test; when sparse expected cell counts were present, Fisher’s exact test with Monte Carlo estimation based on 10,000 samples was used. Mann–Whitney U tests were used for two-group comparisons of continuous variables, whereas Kruskal–Wallis tests with Bonferroni-adjusted post-hoc pairwise comparisons were used for comparisons across healthcare specialties.

A multivariable multinomial logistic regression model was fitted with PA level (low, moderate, and high) as the dependent variable, using high PA as the reference outcome. Healthcare specialty, academic year, and sex were included simultaneously as predictors. Medicine and male sex were used as reference categories. Academic year was entered as a continuous covariate in the primary model to improve model parsimony and estimate stability because relatively few participants were enrolled in the fifth and sixth years. A sensitivity analysis treating academic year as a categorical variable produced substantively similar findings. An additional sensitivity analysis treated total MET-minutes/week as a continuous outcome after natural-log transformation [ln(MET-minutes/week + 1)]. A two-sided *p*-value <0.05 was considered statistically significant.

Ethical approval was obtained from the Institutional Review Board of Hebron, electronic informed consent was secured from all participants, and anonymity and confidentiality were maintained throughout the study in accordance with the Declaration of Helsinki and STROBE guidelines.

## Results

### Participant characteristics

The study included 408 healthcare students from various academic programs and training years in different regions of the West Bank, of whom 88.0% (*n* = 359) were from the Southern West Bank. The majority of participants were female (69.4%, *n* = 283), while males accounted for 30.6% (*n* = 125). The median age was 21 years (IQR: 20–22). Most participants were single (93.4%, *n* = 381). Students in years 2–4 represented 82.1% of the sample (*n* = 335). Nursing students constituted the largest specialty group (39.0%, *n* = 159), followed by medical students (36.0%, *n* = 147). Overall, 76.5% (*n* = 312) reported engaging in at least one type of physical activity on a weekly basis, whereas 23.5% (*n* = 96) reported no weekly physical activity. Detailed sociodemographic and academic characteristics of the participants are presented in Table 1.

**Table 1:** Sociodemographic characteristics among healthcare students.

Parameters	Frequency (*n*)	Percentage (%)
Sex		
Females	283	69.4
Males	125	30.6
Year of study		
First year	49	12
Second year	103	25.2
Third year	82	20.1
Fourth year	150	36.8
Fifth year	14	3.4
Sixth year	10	2.5
Specialty		
Medicine	147	36
Nursing	159	39
Physical therapy	35	8.6
Occupational therapy	26	6.4
Laboratories	18	4.4
Other healthcare ieldsf	23	5.6
Region of residence		
Southern West Bank	359	88
Middle West Bank	26	6.4
Northern West Bank	23	5.6
Marital status		
Single	381	93.4
Married	27	6.6
Do you do any type of PA on weekly basis?		
Yes	312	76.5
No	96	23.5

### Prevalence of physical activity

The most commonly reported type of PA was walking (63.0%, *n* = 257), followed by running (16.2%, *n* = 66) and gym/bodybuilding (15.0%, *n* = 61). Participants could report more than one type of physical activity; therefore, percentages do not sum to 100%. The distribution of reported activity types is presented in Table 2.

**Table 2:** Types of physical activity reported by participants (*n* = 408).

Type of physical activity	*n* (%)
Walking	257 (63.0%)
Running	66 (16.2%)
Gym/Bodybuilding	61 (15.0%)
Football	31 (7.6%)
Swimming	20 (4.9%)
Volleyball	10 (2.5%)
Other physical activities	10 (2.5%)

Participants could select more than one type of physical activity; therefore, percentages do not sum to 100%. Percentages were calculated using the total sample (*n* = 408) as the denominator.

Potential barriers to physical activity were assessed among all participants. Lack of time was the most frequently reported challenge to physical activity in the total sample (65.4%, *n* = 267), followed by unwillingness to engage in physical activity (27.2%, *n* = 111), lack of facilities (23.3%, *n* = 95), injury (4.9%, *n* = 20), and other reasons (3.2%, *n* = 13). When descriptively stratified according to self-reported weekly physical activity, lack of time was reported by 65.4% (*n* = 204) of participants who engaged in weekly PA and 65.6% (*n* = 63) of those who did not. Unwillingness was reported by 22.1% (*n* = 69) and 43.8% (*n* = 42), respectively, while lack of facilities was reported by 21.8% (*n* = 68) and 28.1% (*n* = 27), respectively. Injury was reported by 5.4% (*n* = 17) and 3.1% (*n* = 3), respectively, and other reasons by 3.2% (*n* = 10) and 3.1% (*n* = 3), respectively. Figure 1 illustrates the distribution of reported challenges in the total sample and according to self-reported weekly PA status.

**Figure 1:**
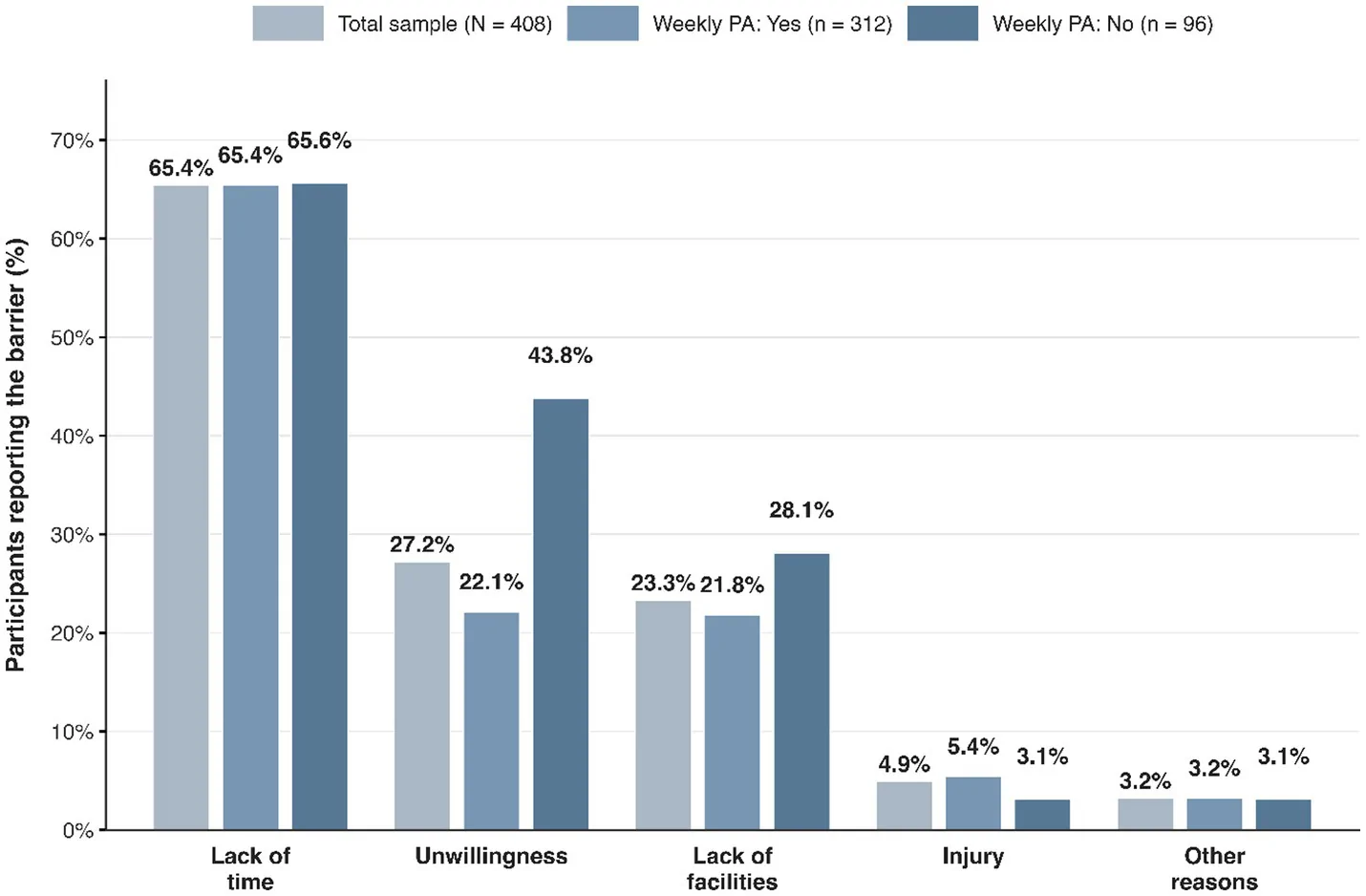
Potential barriers to physical activity in the total sample and according to self-reported weekly physical activity status. Participants could report more than one barrier; therefore, percentages do not sum to 100%.

### Physical activity patterns in the study sample

Across the GPAQ domains, walking or cycling for transport was the most commonly reported activity, with 77.7% (*n* = 317) of participants engaging in active transport for at least 10 continuous minutes, followed by moderate-intensity work-related activity (75.2%, *n* = 307), moderate-intensity leisure activity (64.2%, *n* = 262), vigorous-intensity work-related activity (61.0%, *n* = 249), and vigorous-intensity leisure activity (53.4%, *n* = 218). Among participants reporting activity within each domain, active transport was performed for a median of 5 days per week (IQR: 3–6) and 1 h per day (IQR: 1–3). Vigorous- and moderate-intensity work activities were both performed for a median of 3 days per week, with median durations of 2 h per day. Vigorous-intensity leisure activity was performed for a median of 2 days per week (IQR: 1–3), whereas moderate-intensity leisure activity was performed for a median of 3 days per week (IQR: 2–4). The median sedentary time in the total sample was 5 h per day (IQR: 3–8). Detailed domain-specific activity patterns are presented in Table 3.

**Table 3:** Physical activity patterns across GPAQ domains among participants.

Physical activity domain	Participants reporting activity, *n* (%)	Days/week, Median (IQR)	Hours/day, Median (IQR)
Vigorous-intensity work	249 (61.0)	3 (2–4)	2 (1–3)
Moderate-intensity work	307 (75.2)	3 (2–5)	2 (1–3)
Walking/cycling for transport	317 (77.7)	5 (3–6)	1 (1–3)
Vigorous-intensity leisure	218 (53.4)	2 (1–3)	2 (1–2)
Moderate-intensity leisure	262 (64.2)	3 (2–4)	2 (1–3)
Sedentary time	—	—	5 (3–8)

Activity frequency and duration are summarized only among participants reporting activity in the corresponding GPAQ domain. Sedentary time is summarized for the entire sample (*n* = 408). IQR, interquartile range.

The weekly frequency of activity varied across GPAQ domains. Overall, 39.0% (*n* = 159) of participants reported no vigorous-intensity work activity, while 17.6% (*n* = 72) reported engaging in such activity on 2 days per week and 15.2% (*n* = 62) on 3 days per week. For moderate-intensity work activity, 24.8% (*n* = 101) reported 0 days per week, whereas 21.1% (*n* = 86) reported 2 days per week. Active transport showed a different pattern, with 26.2% (*n* = 107) reporting walking or cycling for transport on 5 days per week and 18.1% (*n* = 74) on all 7 days. Nearly half of participants (46.6%, *n* = 190) reported no vigorous-intensity leisure activity, while 35.8% (*n* = 146) reported no moderate-intensity leisure activity. The complete distribution of weekly activity frequency across the five GPAQ domains is presented in Table 4.

**Table 4:** Days spent doing different types of PA in a typical week.

Physical activity domain	0 days *n* (%)	1 day *n* (%)	2 days *n* (%)	3 days *n* (%)	4 days *n* (%)	5 days *n* (%)	6 days *n* (%)	7 days *n* (%)
Vigorous-intensity work	159 (39.0)	37 (9.1)	72 (17.6)	62 (15.2)	34 (8.3)	23 (5.6)	11 (2.7)	10 (2.5)
Moderate-intensity work	101 (24.8)	30 (7.4)	86 (21.1)	65 (15.9)	49 (12.0)	44 (10.8)	11 (2.7)	22 (5.4)
Walking/cycling for transport	91 (22.3)	14 (3.4)	25 (6.1)	43 (10.5)	39 (9.6)	107 (26.2)	15 (3.7)	74 (18.1)
Vigorous-intensity leisure	190 (46.6)	60 (14.7)	59 (14.5)	53 (13.0)	20 (4.9)	17 (4.2)	5 (1.2)	4 (1.0)
Moderate-intensity leisure	146 (35.8)	46 (11.3)	80 (19.6)	61 (15.0)	29 (7.1)	31 (7.6)	8 (2.0)	7 (1.7)

Percentages were calculated using the total sample (*n* = 408) as the denominator. Participants reporting no activity in a specific domain are represented in the 0-days category.

According to the WHO Global Physical Activity Questionnaire (GPAQ) scoring criteria, participants were classified into low, moderate, and high physical activity levels based on their total MET-minutes/week. Overall, 68.1% (*n* = 278) were classified as having a high physical activity level, 22.5% (*n* = 92) as moderate, and 9.3% (*n* = 38) as low (Figure 2).

**Figure 2:**
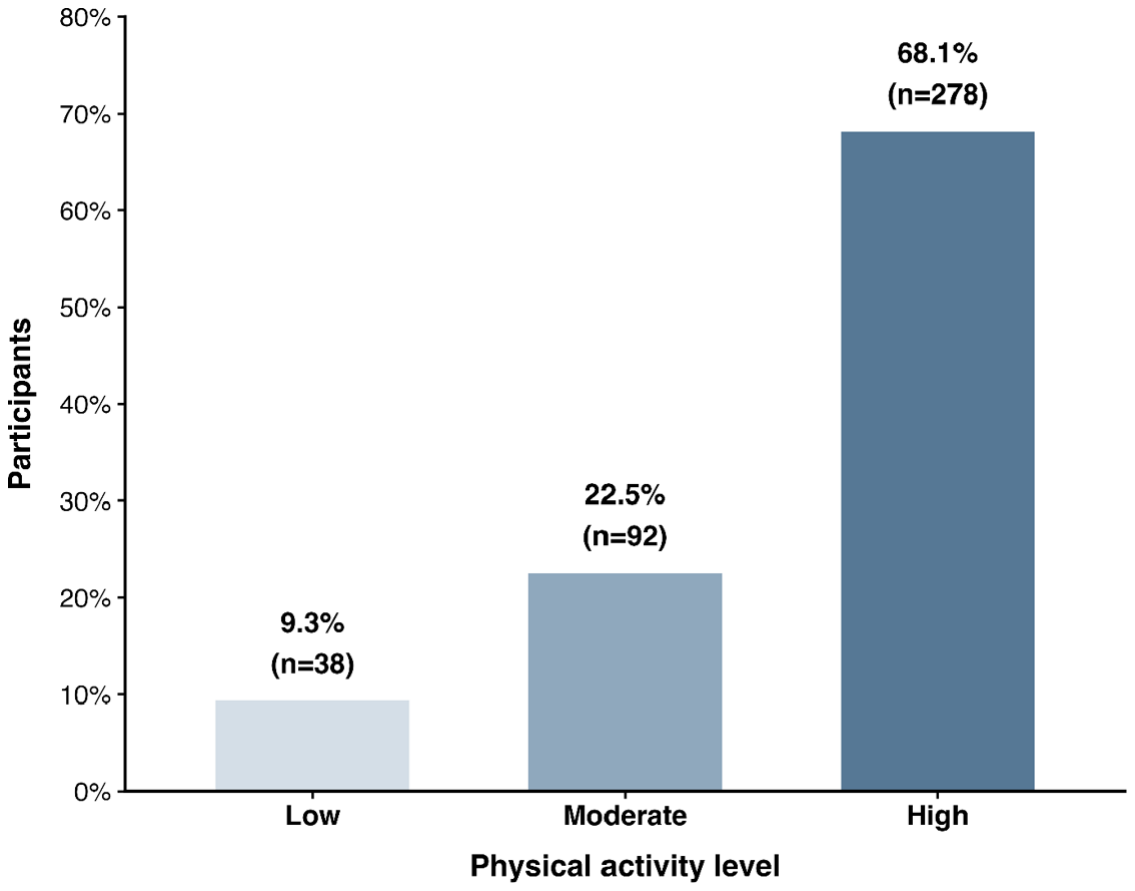
Distribution of WHO GPAQ physical activity levels among healthcare students (*n* = 408).

Physical activity level did not differ significantly by sex (*χ*^2^(2) = 1.002, *p* = 0.606). However, total physical activity expressed as MET-minutes/week was higher among males, with a median of 6,720 MET-min/week (IQR: 2,376–10,320), compared with 4,320 MET-min/week (IQR: 2,160–9,360) among females (Mann–Whitney U = 15,341, *p* = 0.033). In domain-specific comparisons, males showed higher levels of vigorous-intensity work activity (*p* = 0.012) and vigorous-intensity leisure activity (*p* = 0.001), whereas no significant sex differences were observed for moderate-intensity work activity (*p* = 0.971), active transport (*p* = 0.745), or moderate-intensity leisure activity (*p* = 0.154).

A Monte Carlo Fisher’s exact test showed a statistically significant association between healthcare specialty and WHO GPAQ physical activity level (*p* < 0.001). As visualized in Figure 3, noticeable variations in PA levels were observed across healthcare specialties, with the proportion classified as having a high PA level ranging from 50.3% among medical students to 84.6% among occupational therapy students. A Kruskal–Wallis test was conducted to assess whether total PA and domain-specific PA time differed significantly across specialties. Significant differences were observed for total PA (H(5) = 32.550, *p* < 0.001), vigorous-intensity work activity (H(5) = 36.695, *p* < 0.001), moderate-intensity work activity (H(5) = 36.458, *p* < 0.001), and moderate-intensity leisure activity (H(5) = 17.883, *p* = 0.003). In contrast, no statistically significant differences were found for active transportation (H(5) = 11.053, *p* = 0.050) or vigorous-intensity leisure activity (H(5) = 10.691, *p* = 0.058). Bonferroni-adjusted post-hoc pairwise comparisons showed that medical students had significantly lower total PA than nursing students (adjusted *p* < 0.001) and physical therapy students (adjusted *p* = 0.006). For vigorous-intensity work activity, medical students differed significantly from nursing students (adjusted *p* < 0.001) and occupational therapy students (adjusted *p* = 0.012). For moderate-intensity work activity, medical students differed significantly from both nursing and physical therapy students (both adjusted *p* < 0.001). For moderate-intensity leisure activity, the only significant pairwise difference was observed between medical and nursing students (adjusted *p* = 0.002). Table 5 presents total PA and domain-specific activity time across healthcare specialties.

**Figure 3:**
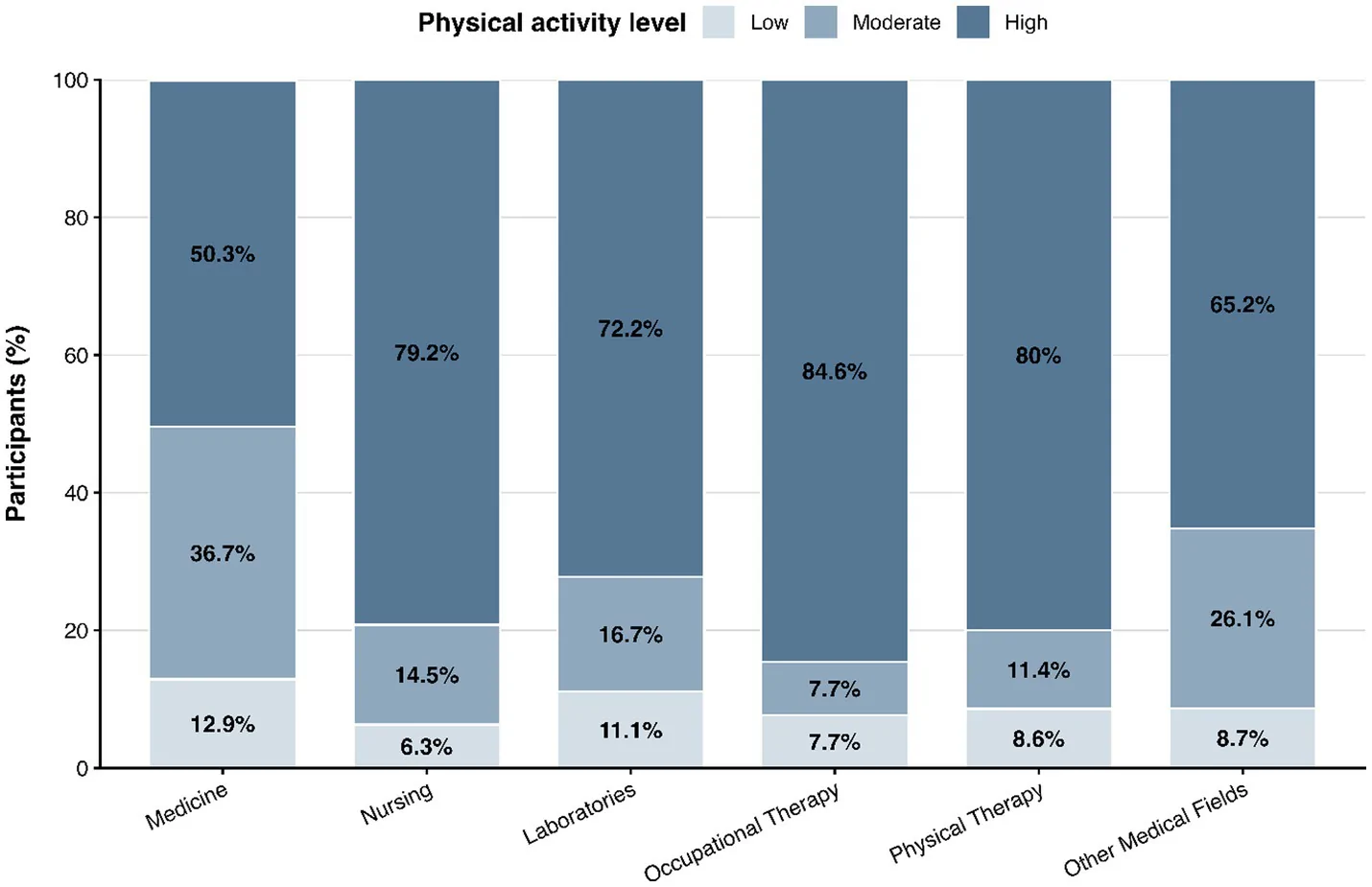
Distribution of WHO GPAQ physical activity levels across healthcare specialties.

**Table 5:** Total physical activity and domain-specific activity time across healthcare specialties.

Variable	Medicine	Nursing	Laboratories	Occupational therapy	Physical therapy	Other healthcare fields	*p*-value
Total PA, ×10^3^ MET-min/week	3.12 (1.20–6.72)	6.72 (3.68–10.80)	4.86 (2.40–10.14)	5.16 (3.33–10.62)	7.12 (3.12–12.00)	5.16 (1.92–9.36)	**<0.001**
Vigorous-intensity work, h/day	0 (0–1)	1 (0.17–2)	0.63 (0–1.25)	1 (0.23–3)	1 (0–2)	0.50 (0–3)	**<0.001**
Moderate-intensity work, h/day	1 (0–2)	2 (1–3)	2 (0.96–2.25)	2 (0.88–2.25)	3 (2–3)	2 (0–3)	**<0.001**
Walking/cycling for transport, h/day	1 (0.25–2)	1 (0.33–2)	2 (0.83–3)	0.50 (0–2)	1.50 (0.33–3)	1 (0–2)	0.050
Vigorous-intensity leisure, h/day	0 (0–1)	1 (0–2)	0.25 (0–1.25)	0.25 (0–2)	0 (0–1.50)	0 (0–1.50)	0.058
Moderate-intensity leisure, h/day	0.50 (0–2)	1 (0–3)	1 (0–2)	1 (0–2)	1 (0–2)	0.25 (0–1)	**0.003**

Data are presented as median (IQR). Total physical activity is expressed in thousands of MET-minutes/week (×10^3^ MET-min/week). Overall differences across healthcare specialties were assessed using the Kruskal–Wallis test. Domain-specific activity times are presented in hours/day and include all participants within each specialty, including zero values for participants reporting no activity in the corresponding domain. A *p*-value <0.05 was considered statistically significant. Bold values mean a statistically significant *p*-value.

Physical activity levels showed descriptive variation across academic years, as illustrated in Figure 4. The proportion of students classified as having high PA was 77.6% in the first year, 69.9% in the second year, 63.4% in the third year, 70.0% in the fourth year, 50.0% in the fifth year, and 40.0% in the sixth year. The corresponding proportions classified as having moderate PA were 12.2, 21.4, 25.6, 21.3, 42.9, and 50.0%, respectively, while the proportion with low PA remained relatively small across all academic years. Because the fifth- and sixth-year groups included relatively few participants (*n* = 14 and *n* = 10, respectively), these descriptive differences should be interpreted cautiously.

**Figure 4:**
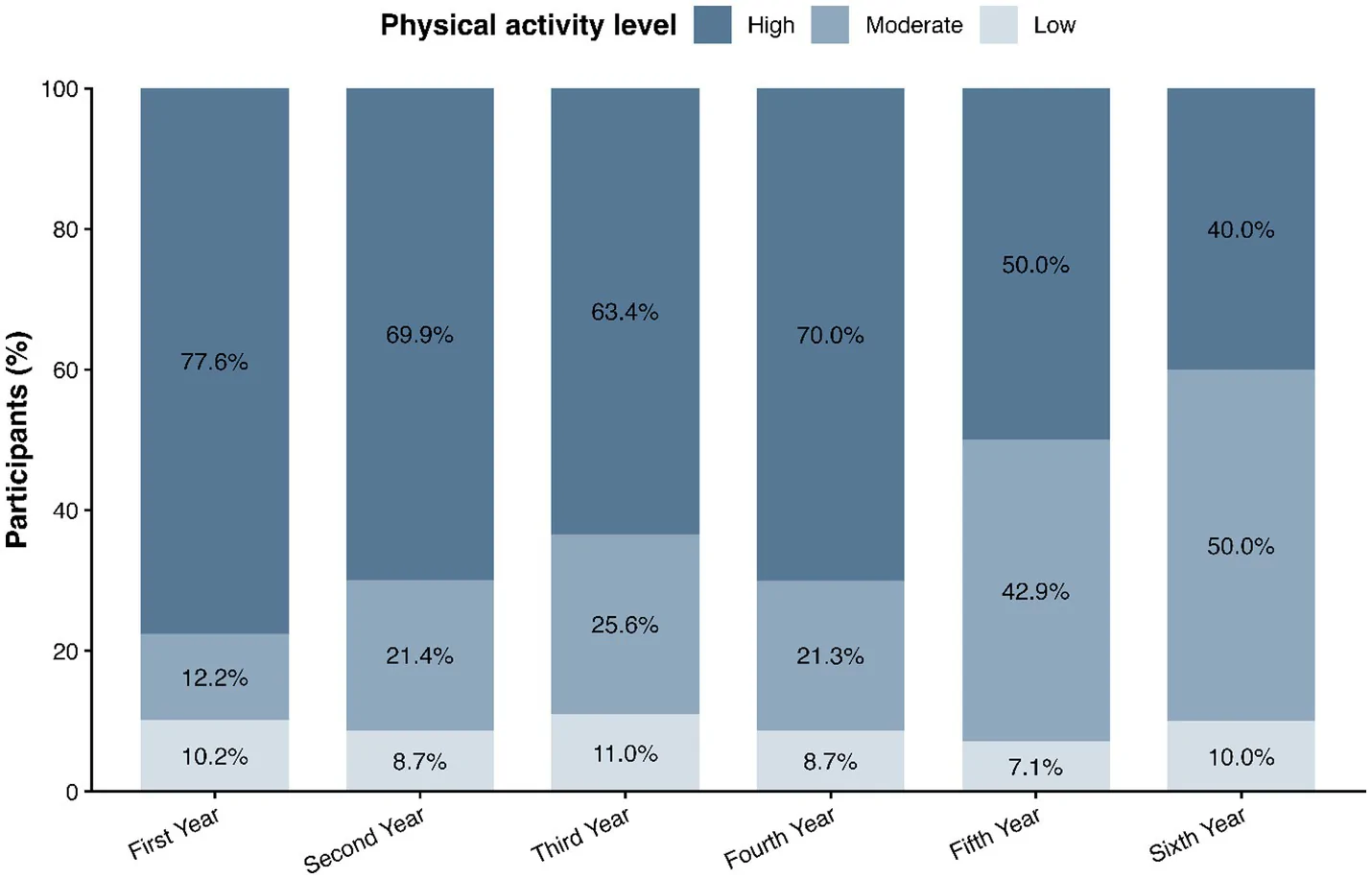
Distribution of WHO GPAQ physical activity levels across academic years.

In the multivariable multinomial logistic regression model, with high PA as the reference outcome category, the overall model was statistically significant (likelihood-ratio *χ*^2^(14) = 40.744, *p* < 0.001). Healthcare specialty was independently associated with PA level (likelihood-ratio *χ*^2^(10) = 34.374, *p* < 0.001), whereas academic year (*p* = 0.487) and sex (*p* = 0.580) were not. Compared with medical students, nursing students had significantly lower odds of being in the low rather than high PA category (aOR = 0.30, 95% CI: 0.13–0.68, *p* = 0.004). For moderate versus high PA, nursing students (aOR = 0.27, 95% CI: 0.15–0.47, *p* < 0.001), occupational therapy students (aOR = 0.13, 95% CI: 0.03–0.58, *p* = 0.007), and physical therapy students (aOR = 0.21, 95% CI: 0.07–0.65, *p* = 0.007) had significantly lower odds of being moderately rather than highly active compared with medical students. Academic year and sex were not significantly associated with either outcome comparison (Table 6).

**Table 6:** Multivariable multinomial logistic regression analysis of factors associated with physical activity level.

Predictor	Category	Low vs High, aOR (95% CI)	*p*-value	Moderate vs High, aOR (95% CI)	*p*-value
Specialty	Nursing	**0.30 (0.13–0.68)**	**0.004**	**0.27 (0.15–0.47)**	**<0.001**
	Laboratories	0.53 (0.11–2.58)	0.430	0.33 (0.09–1.24)	0.102
	Occupational therapy	0.33 (0.07–1.53)	0.156	**0.13 (0.03–0.58)**	**0.007**
	Physical therapy	0.39 (0.10–1.45)	0.159	**0.21 (0.07–0.65)**	**0.007**
	Other healthcare fields	0.47 (0.10–2.29)	0.349	0.61 (0.22–1.72)	0.347
	Medicine	Reference	—	Reference	—
Academic year	Per one-year increase	0.92 (0.69–1.24)	0.583	1.11 (0.89–1.37)	0.357
Sex	Female	1.51 (0.68–3.38)	0.312	1.09 (0.64–1.87)	0.754
	Male	Reference	—	Reference	—

High physical activity was the reference outcome category. Adjusted odds ratios (aORs) were obtained from a multivariable multinomial logistic regression model including healthcare specialty, academic year, and sex simultaneously. Medicine and male sex were used as the reference categories. Academic year was entered as a continuous covariate; therefore, its aOR represents the change in odds associated with each one-year increase in academic year. An aOR <1 indicates lower odds of being in the low or moderate PA category rather than the high PA category relative to the corresponding reference group. Bold values mean a statistically significant *p*-value.

Sensitivity analyses supported the robustness of these findings. Treating academic year as a categorical rather than continuous variable did not materially alter the results. Similarly, when total MET-minutes/week was analyzed as a continuous outcome after natural-log transformation, healthcare specialty remained significantly associated with total PA (*F*(5,400) = 3.014, *p* = 0.011), whereas academic year (*p* = 0.402) and sex (*p* = 0.194) remained non-significant.

## Discussion

The present study found generally high levels of physical activity among healthcare students, with 90.7% classified as having moderate or high physical activity and 68.1% classified as highly active according to WHO GPAQ criteria. Physical activity differed significantly across healthcare specialties, with nursing and physical therapy students showing higher total physical activity than medical students in post-hoc comparisons. In the adjusted analysis, healthcare specialty remained significantly associated with physical activity level, whereas sex and academic year were not independently associated. Lack of time was the most commonly reported challenge or constraint to physical activity to physical activity, reported by 65.4% of participants.

Although 76.5% of healthcare students reported engaging in at least one type of physical activity during a typical week, this proportion was lower than the 90.7% classified as having moderate or high physical activity according to WHO GPAQ criteria. These findings reflect different aspects of physical activity. The weekly PA question reflects students’ self-reported perception of whether they engaged in physical activity, whereas the GPAQ classification considers the frequency, duration, and intensity of activity across different domains. This discrepancy may suggest that some students may not fully recognize what constitutes sufficient or regular physical activity according to WHO recommendations.

In Southern Thailand, 49.5% of medical students met the recommended level of PA based on GPAQ assessment (17). Similarly, among medical students at the University of Khartoum, Sudan, 40.5% achieved the WHO-recommended level of PA (24). In contrast, an Australian study using the International Physical Activity Questionnaire-Short Form reported that 92% of pre-clinical medical students were classified as moderately or highly active (25). Comparisons across these studies should be interpreted cautiously because of differences in study populations, PA assessment instruments, and classification criteria.

Physical activity level did not differ significantly between male and female students in the present study. However, this similarity in the categorical GPAQ classification did not indicate identical activity patterns between sexes. Males had significantly higher total PA than females, with median values of 6,720 and 4,320 MET-min/week, respectively (*p* = 0.033). Males also had significantly higher vigorous-intensity work activity (*p* = 0.012) and vigorous-intensity leisure activity (*p* = 0.001), whereas no significant sex differences were observed in moderate-intensity work activity, active transport, or moderate-intensity leisure activity. In Southern Thailand, male medical students were more likely than females to meet recommended PA levels (17). Similarly, a study among medical students at the University of Khartoum reported higher vigorous-intensity and total PA among males than females (24). Thus, although sex was not associated with the broad low, moderate, and high PA classification in our sample, differences were evident in the overall volume and vigorous-intensity components of physical activity.

Walking was the most commonly reported type of PA in the present study, reported by 63.0% of participants. This finding is consistent with a study of healthcare students at King Saud University, where walking was also the most commonly reported physical activity, reported by 42.7% of participants (18). Similarly, a study among medical students at Al-Jouf University reported that 33.2% of participants achieved the specified health-enhancing walking criterion (19). Evidence from Poland has also indicated that walking is among the most frequently reported forms of physical activity among university students (26). Walking may represent a particularly accessible form of physical activity for university students because it can be incorporated into routine daily activities, including commuting to campus and moving between academic or clinical settings, while requiring little or no specialized equipment or facilities.

Lack of time was the most frequently reported perceived challenge to PA in the present study, reported by 65.4% of the total sample. Importantly, lack of time was reported at almost identical proportions among participants who engaged in weekly PA and those who did not (65.4 and 65.6%, respectively). Therefore, lack of time should be interpreted as a broadly perceived constraint among healthcare students rather than a barrier that specifically prevents participation in physical activity. In contrast, unwillingness to engage in PA was descriptively more common among participants reporting no weekly PA than among those reporting weekly PA (43.8% vs. 22.1%). Among healthcare students at King Saud University, lack of time was the primary reason for inactivity, reported by 51.4% of participants (18). Similarly, among medical students at Al-Jouf University, time limitations were the most frequently reported barrier to PA (19). A study among medical students at the University of Khartoum also identified lack of time as a major barrier, reported by 43.8% of participants (24). Among university students in Saudi Arabia, time restrictions were likewise the most common reason for not practicing exercise during college (27). Consistent with these findings, a systematic review published in 2022 identified lack of time as one of the most frequently reported barriers to physical activity among high school and university students (28), Collectively, these findings indicate that time constraints are a recurrent perceived barrier across university and healthcare-student populations, although in the present study they were reported similarly by students with and without weekly PA.

The weekly frequency of PA varied considerably across GPAQ domains in the present study. Overall, 39.0% of participants reported no vigorous-intensity work activity, while 17.6 and 15.2% reported engaging in this activity on two and three days per week, respectively. For moderate-intensity work activity, 21.1% of the total sample reported participation on two days per week. Active transport showed a different pattern, with 26.2% reporting walking or cycling for transport on five days per week and 18.1% doing so on all seven days. Nearly half of the participants (46.6%) reported no vigorous-intensity leisure activity, whereas 35.8% reported no moderate-intensity leisure activity. A study among healthcare students at King Saud University similarly demonstrated variation in weekly activity frequency across GPAQ domains; among students reporting activity in the corresponding domains, 25.4% performed vigorous-intensity work activity on three days per week, 22.3% performed moderate-intensity work activity on three days per week, and 21.9% used active transport on five days per week (18). Although both studies used the GPAQ, direct comparison of these percentages should be made cautiously because the Saudi study summarized weekly frequency among participants reporting activity within each domain, whereas the present study calculated the frequency distribution using the total sample as the denominator.

The median sedentary time in the present study was 5 h per day (IQR: 3–8). Among participants reporting activity within the corresponding GPAQ domains, the median duration was 2 h/day for vigorous-intensity work, 2 h/day for moderate-intensity work, 1 h/day for active transport, and 2 h/day for both vigorous- and moderate-intensity leisure activity. Among healthcare students at King Saud University, mean sedentary time was 5.37 h/day, while mean vigorous-intensity work activity was 1.5 h/day (18). In comparison, medical students at the University of Khartoum reported a mean sedentary time of 7.33 h/day (24). Direct comparisons of activity duration across these studies should be interpreted cautiously because of differences in study populations and statistical summaries, particularly because the present study reports medians and IQRs for non-normally distributed activity variables, whereas the comparison studies primarily reported means and standard deviations. Differences in the composition of healthcare programs and academic settings may also contribute to variation in reported physical activity and sedentary patterns.

Healthcare specialty was significantly associated with PA level in the present study. Medical students had the lowest proportion classified as having high PA (50.3%), compared with 79.2% among nursing students, 72.2% among laboratory students, 84.6% among occupational therapy students, 80.0% among physical therapy students, and 65.2% among students from other healthcare fields. Bonferroni-adjusted post-hoc comparisons further showed that medical students had significantly lower total PA than nursing and physical therapy students. Medical students also had lower vigorous-intensity work activity than nursing and occupational therapy students, lower moderate-intensity work activity than nursing and physical therapy students, and lower moderate-intensity leisure activity than nursing students. In contrast, active transportation and vigorous-intensity leisure activity did not differ significantly across healthcare specialties.

The multivariable analysis further supported specialty-related differences in PA. Compared with medical students, nursing students had lower odds of being in the low rather than high PA category, while nursing, occupational therapy, and physical therapy students had lower odds of being in the moderate rather than high PA category. This pattern differs from the findings of Blake et al., who reported that nursing students were less active than medical students and that a greater proportion of nursing students failed to meet recommended PA levels (15). It also contrasts with a study of nursing students at the University of California, Los Angeles, in which baccalaureate nursing students were predominantly represented in the low-PA category (29). These contrasting findings suggest that specialty-related PA patterns may vary across academic and institutional contexts. Differences in curriculum structure, clinical schedules, and opportunities for activity may potentially contribute to such variation, although these factors were not directly evaluated in the present study.

Previous studies have reported heterogeneous findings regarding the relationship between academic year and PA. In a multicenter study of medical students from the Western Balkans, significant year-related differences were observed at individual institutions but in opposite directions: at the University of Ljubljana, students in years 4–6 were more likely to report no regular PA than those in years 1–3, whereas at the University of Zenica, students in years 1–3 were more frequently inactive than those in years 4–6 (30). However, when the entire study sample was considered, no significant difference in PA was observed according to year of study (30). In a large study of German university students, higher-year students reported slightly greater PA than first-year students; although the difference was statistically significant, the effect size was negligible (partial η^2^ = 0.001) (31). In the present study, academic year was not independently associated with PA level in the multivariable multinomial model (*p* = 0.487). Although descriptive variation in PA classification was observed across academic years, particularly among fifth- and sixth-year students, these groups included relatively few participants (*n* = 14 and *n* = 10, respectively), warranting cautious interpretation. The absence of an independent association with academic year was further supported by sensitivity analyses using categorical coding of academic year and log-transformed total MET-minutes/week, for which academic year also remained non-significant (*p* = 0.402). Overall, these findings do not support a consistent independent association between academic year and PA in the present sample after accounting for healthcare specialty and sex.

This study has several limitations that should be considered when interpreting the findings. First, the cross-sectional design precludes establishing causal relationships between academic characteristics, perceived barriers, and physical activity (PA). Second, PA and perceived barriers were assessed using self-reported measures, which may be subject to recall and social desirability bias. Third, convenience sampling may have introduced selection bias and limited the representativeness of the sample. Notably, 88.0% (*n* = 359) of participants were recruited from the Southern West Bank, resulting in limited representation of students from central and northern regions and potentially restricting the generalizability of the findings to healthcare students across the West Bank.

The sample was also unevenly distributed across academic years, with students in Years 2–4 comprising most participants, while Years 5 and 6 were substantially underrepresented (3.4 and 2.5%, respectively). This imbalance may have reduced the statistical power to detect differences in PA across academic years and may partly explain the lack of a significant association between academic year and PA. In addition, the participating specialties differ in program structure and duration; therefore, the same academic year may not correspond to the same stage of professional training across specialties. For example, a fourth-year nursing student may be in the final year of training, whereas a fourth-year medical student may still be in the middle of a longer program. Consequently, comparisons based solely on academic year may not fully capture differences in students’ training stages.

Furthermore, some specialty and academic-year subgroups were relatively small, particularly those in Years 5 and 6, which may have reduced the precision of subgroup-specific estimates. These sampling and subgroup imbalances should therefore be considered when interpreting the observed associations between specialty, academic year, and PA. Future studies should employ stratified or proportionate sampling across geographic regions, specialties, and academic years to achieve more balanced representation and improve the generalizability and precision of the findings.

## Conclusion

This study found that most healthcare students had moderate or high PA levels, although PA patterns varied significantly across healthcare specialties. Medical students showed comparatively lower total PA than nursing and physical therapy students, whereas academic year and sex were not independently associated with PA level in multivariable analysis. Walking was the most commonly reported form of PA, and lack of time was the leading perceived challenge. These findings support consideration of accessible, specialty-sensitive opportunities for physical activity that can be integrated into demanding academic and clinical schedules.

## Data Availability

The raw data supporting the conclusions of this article will be made available by the authors, without undue reservation.
